# Hangeshashinto-Associated Mesenteric Phlebosclerosis and Highly Atypical Adenoma Requiring Laparoscopic Right Hemicolectomy

**DOI:** 10.3390/diagnostics14050565

**Published:** 2024-03-06

**Authors:** Ryo Nishiwaki, Yasuhiro Inoue, Masataka Sugao, Natsuko Sugimasa, Tetsuya Hamaguchi, Midori Noji, Kenji Takeuchi, Yoshiyuki Ito, Toshio Kato, Taro Yasuma, Corina N. D’Alessandoro-Gabazza, Esteban C. Gabazza, Ichiro Imoto

**Affiliations:** 1 Department of Surgery, Doshinkai Tohyama Hospital, Tsu 514-0043, Japan; 2 Department of Immunology, Mie University Faculty and Graduate School of Medicine, Tsu 514-8507, Japan; 3 Department of Internal Medicine, Doshinkai Tohyama Hospital, Tsu 514-0043, Japan; 4 Digestive Endoscopy Center, Doshinkai Tohyama Hospital, Tsu 514-0043, Japan

**Keywords:** mesenteric phlebosclerosis, Hangeshashinto, endoscopic mucosal resection, highly atypical adenoma

## Abstract

Mesenteric phlebosclerosis is a rare ischemic colonic disorder caused by impaired venous drainage. Its prevalence is higher in East Asia, where herbal medicine is widely used. Treatment remains controversial. A 76-year-old woman who had taken Hangeshashinto, an herbal medicine, for 11 years was admitted for endoscopic treatment of high-grade dysplasia in the ascending colon. She had diarrhea and mesenteric phlebosclerosis diagnosed by abdominal computed tomography at age 71. At age 75, small polyps were detected in the ascending colon. A subsequent study revealed an increase in polyp size to 15 mm. Endoscopic mucosal resection failed to remove the lesion. A biopsy showed high-grade dysplasia with possible colon cancer risk. Conservative therapy did not improve mesenteric phlebosclerosis-related diarrhea; therefore, a laparoscopic right hemicolectomy was performed. Intraoperatively, the cecum was adherent to the abdominal wall and the right ovary. The specimen showed high-grade dysplasia in the mucosa and severe submucosal fibrosis. No metastasis was observed. This case shows the link between mesenteric phlebosclerosis and high-grade dysplasia in the ascending colon. Endoscopic mucosal resection was unsuccessful in removing the tumor. Endoscopic submucosal dissection was an alternative, but its safety in mesenteric phlebosclerosis-affected colonic segments remains uncertain. A laparoscopic right hemicolectomy was performed.

**Figure 1:**
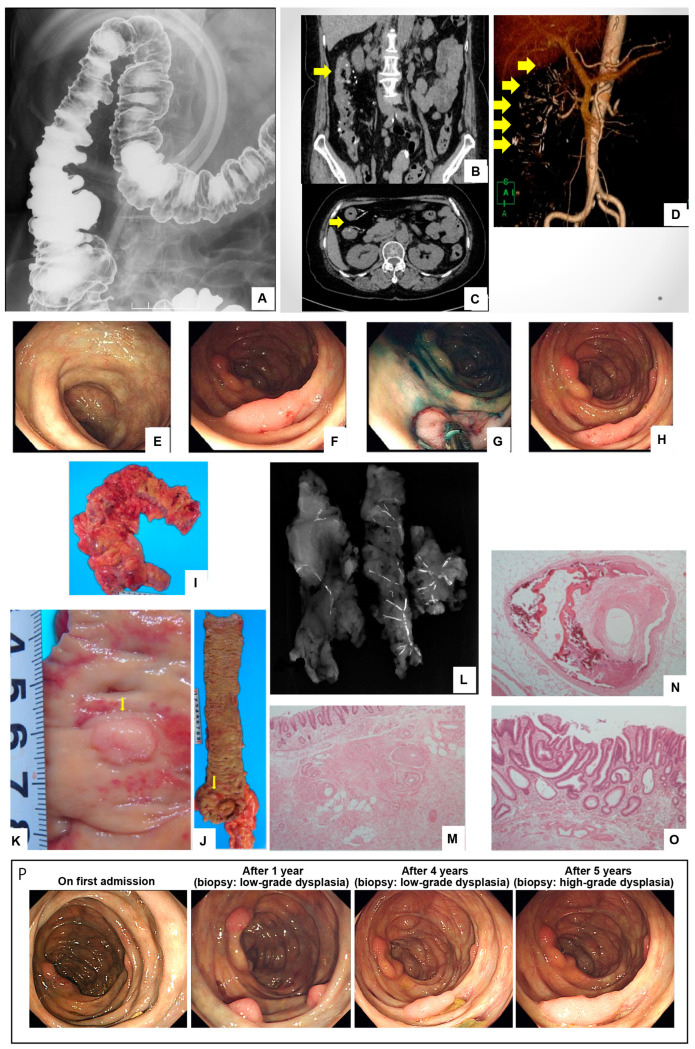
High-grade dysplasia coexisting with mesenteric phlebosclerosis (MPh) in a patient with a history of Hangeshashinto use and refractory diarrhea, who underwent laparoscopic right hemicolectomy. A 76-year-old woman with a medical history of hypertension, cesarean section, and type C liver cirrhosis secondary to blood transfusion was admitted to our hospital to undergo endoscopic mucosal resection (EMR) of a high-grade dysplasia in the ascending colon. She had been taking the herbal medicine called Hangeshashinto for lower leg edema for 11 years since she was 62. At the age of 71, she developed diarrhea and underwent various tests, including colonoscopy and abdominal computed tomography (CT), and was diagnosed with MPh. She subsequently discontinued Hangeshashinto, the suspected causative agent of MPh, and underwent an annual colonoscopy follow-up. At the age of 75, a colonoscopy revealed multiple polyps of approximately 5 mm in diameter on and near the ileocecal valve. One year later, a significant increase in the size of one polyp prompted the decision to proceed with EMR. On admission, she was 140 cm tall, weighed 44 kg, had a body mass index of 22, a blood pressure of 156/67 mmHg, and a pulse rate of 63/min. No superficial lymph nodes or abdominal tenderness were palpable. The blood tests showed no anemia or inflammation, but moderate renal impairment (creatinine 0.95 mg/dl, estimated glomerular filtration rate 45.3 mL/min) was noted. Tumor markers were within normal limits (CEA 5.0 ng/mL, CA19-9 35.6 U/mL). Abdominal plain radiography showed no obvious abnormalities, including colonic wall calcification. A double-contrast barium enema study revealed poor haustration near the hepatic flexure of the ascending colon, as well as impaired wall distensibility, mucosal irregularity, serration of the lumen, and thumbprinting (**A**). Abdominal computed tomography (CT) demonstrated diffuse wall thickening from the ascending colon to the right transverse colon. Moreover, multiple linear and punctate calcifications (yellow arrow in **B**) were observed within the thickened colon wall (yellow arrow in **C**) and along the course of the vessels from the mesenteric margin to the mesentery (yellow arrows in **D**). A colonoscopy revealed dark purple discoloration of the edematous mucosa, wall thickening, loss of folds, reduced vascular pattern, and partial dilation of submucosal veins (**E**). In addition, several flat elevated lesions were detected in the ascending colon, the largest of which measured approximately 15 mm in size and exhibited a central depression (**F**). A colonoscopy after indigo carmine spraying showed an apparent central depression on the surface of the largest adenoma (**G**). An attempt was made to remove the lesion via endoscopic mucosal resection (EMR); however, a positive non-lifting sign was observed following saline injection (**H**), rendering resection challenging with this technique. A histological examination of biopsy specimens revealed high-grade intraepithelial dysplasia. The malignant transformation could not be definitively excluded, given the inherent risk of carcinogenesis in colorectal adenomas larger than 10 mm [1]. Therefore, we decided to perform a laparoscopic right hemicolectomy for the treatment of the ascending colon tumor and MP. Laparoscopically, the cecum to the hepatic flexure exhibited thickening, sclerosis, and dark purple changes. The surgical intervention was conducted at the root of the ileocolic artery and the right branch of the middle colic artery, ensuring adequate margins to maintain blood flow at the anastomosis. The resected specimen (**I**) displayed a dark purple discoloration of the colonic wall, featuring a flat elevated lesion measuring 13 mm in diameter and two elevated lesions sized 5–8 mm in the ascending colon (yellow arrows in **J**, **K**). Soft-tissue radiography of the resected mesentery revealed thread-like calcified veins (**L**). A pathological examination of MPh disclosed severe fibrous thickening of the submucosa, hyaline degeneration, and calcification of the venous wall, along with partially obliterated vessels (**M**,**N**). The elevated lesion (**K**) was diagnosed as high-grade intraepithelial dysplasia (**O**). A series of representative colonoscopy images illustrating the sequential changes in enlarging polyps is depicted in panel (**P**) from admission up to immediately prior to laparoscopic intervention. There were no postoperative anastomotic leaks or signs of recurrence after 20 months. MPh is a non-obstructive, non-thrombotic, and non-inflammatory chronic sclerosis of the mesenteric vein, with a higher prevalence in the right colon [2]. Koyama et al. first described MPh in 1991 as chronic ischemic colitis with stenosis of the right colon [3]. In 2003, Iwashita et al. proposed a novel disease concept, identifying idiopathic MPh through the study of seven patients exhibiting non-obstructive stenosis or occlusion of the mesenteric veins [2]. While the etiology of MPh remains unclear, prolonged use of herbal medicines has been implicated as a potential risk factor for its development [4,5]. Subsequent reports from Japan have demonstrated a strong association between MPh and the use of herbal medicines, particularly those containing gardenia fruits (*sanshishi* in Japanese) [6,7]. In the present case, the patient had been taking Hangeshashinto, an herbal medicine that does not contain geniposide. There is no report of MPh development due to Hangeshashinto. Ohtsu et al. conducted a nationwide survey that included their own cases to investigate the potential association between herbal medicine and MPh [8]. They identified an association between three components, licorice (glycyrrhizin), omanthus (baicalin), poria cocos, and MPh, in addition to gardenia fruit. Hangeshashinto contains licorice and omanthus. These components are glycosides with beta-glycosidic linkages. Glycosides that reach the lower gastrointestinal tract are metabolized by the beta-glucosidase of intestinal bacteria and absorbed from the right colon as aglycones [9]. The precise mechanism by which aglycones damage the mesenteric venous wall remains unclear. However, it is possible that metabolite products of glycosides promote a release of oxygen reactive species, which may cause tissue injury and abnormal tissue repair. The therapeutic approach for MPh encompasses both conservative management and surgical intervention. In cases involving herbal medicine use, the primary recommendation is the discontinuation of herbal consumption. Conservative treatment for MPh includes gastrointestinal rest and symptomatic therapy. While some reports have demonstrated symptomatic improvement with the combination of aspirin and warfarin [10], the efficacy of antithrombotic drugs in MPh remains unvalidated. In addition, Kohga et al. reported that mesalazine, a medication commonly used for inflammatory bowel disease, was effective in achieving clinical remission of MPh [11]. In cases presenting with severe symptoms, such as bowel obstruction or peritonitis, surgical intervention is usually recommended [4]. However, some cases presenting with bowel obstruction may recover with conservative treatment alone [12]. Therefore, the decision for emergency surgery should be made after careful evaluation [12]. The potential association between colonic mucosa with MPh and cancer remains a topic of debate. In a literature review of 10 cases with concomitant colorectal cancer and MPh, Minami et al. found that 9 out of 10 cases (90%) exhibited cancer in the right colon affected by MPh. However, due to the limited number of cases, further investigation is warranted to conclusively establish a link between MPh and the development of colon cancer. In the current case, a high-grade dysplasia measuring over 10 mm was detected in the right colon with MPh. We initially attempted to remove the lesion using endoscopic mucosal resection (EMR), but the severe fibrosis in the colon’s submucosal layer rendered this technique ineffective. In such instances, endoscopic submucosal dissection (ESD) is typically recommended as the subsequent treatment option in Japan [13]. Kawasaki et al. reported a successful case of ESD for the treatment of transverse colon cancer on a wall affected by MP [14]. However, in two cases, resection of the lesion was not possible with ESD, and surgical intervention was required because of perforation after ESD therapy [15,16]. Consequently, the safety of ESD remains unestablished for colonic epithelial tumors associated with MPh. In this particular case, diarrhea persisted even after discontinuation of Hangeshashinto. Therefore, we opted for a right hemicolectomy to treat the high-grade dysplasia and MPh. In summary, this case involves MPh with characteristic imaging findings associated with prolonged Hangeshashinto use, a herbal medicine from gardenia fruit. Although gardenia fruit is typically identified as the main cause of MPh, other herbal components in Hangeshashinto may also serve as potential causative agents. The relationship between MPh and the development of colon cancer remains unclear; however, regular surveillance is recommended for patients with MPh to monitor potential neoplastic progression. This is especially important for patients associated with atypical adenoma, as in the present case.

## Data Availability

The authors declare that all the data described in this article are available upon reasonable request.
